# Supplementary material for: Genome-Wide Identification and Characterization of Copper Chaperone for Superoxide Dismutase (CCS) Gene Family in Response to Abiotic Stress in Soybean
Source: Int J Mol Sci. 2023 Mar 8;24(6):5154. doi: 10.3390/ijms24065154 (PMC10048983; doi:10.3390/ijms24065154)
Supplement: Supplementary file 1 [file ijms-24-05154-s001.zip › ijms-2238284-supplementary.pdf]

Supplementary Table S1

Supplementary Table S1. List of *GmCCS* genes and their chemical characteristics.

Gene name	Gene locus	Start position	End position	CDS length	MW （kDa）	pI	Subcellular location
GmCCS1	Glyma.03G183300.1	39516238	39518212	540	36.37	8.82	Nucleus, Golgi apparatus
GmCCS2	Glyma.03G242900.1	44076744	44079720	375	15.24	5.59	Nucleus
GmCCS3	Glyma.03G242900.2	44076299	44079720	396	15.24	5.59	Nucleus
GmCCS4	Glyma.03G242900.3	44076299	44079720	351	11.25	6.16	Nucleus
GmCCS5	Glyma.04G197300.1	46933093	6935391	462	36.30	5.65	Nucleus
GmCCS6	Glyma.04G197300.3	4996520	5001001	396	35.03	6.04	Nucleus
GmCCS7	Glyma.05G055000.1	4996520	5001001	462	32.48	5.55	Golgi apparatus
GmCCS8	Glyma.05G055000.2	35001245	35006275	1152	31.55	5.89	Golgi apparatus
GmCCS9	Glyma.05G157900.1	35008421	35010378	486	16.08	8.79	Golgi apparatus
GmCCS10	Glyma.10G112000.1	26763875	26767833	432	8.56	6.19	Cell membrane
GmCCS11	Glyma.10G203200.1	43459433	43462045	507	13.64	4.65	Golgi apparatus,Nucleus
GmCCS12	Glyma.11G091400.1	6922211	6925704	432	17.35	9.23	Golgi apparatus
GmCCS13	Glyma.11G192700.1	26591332	26595068	558	21.65	5.87	Cell membrane,chloroplast
GmCCS14	Glyma.11G192700.2	26591332	26595068	612	20.83	6.03	Cell membrane
GmCCS15	Glyma.11G192700.3	26591332	26595068	621	20.83	6.03	Cell membrane
GmCCS16	Glyma.11G192700.4	26591332	26595068	621	20.89	6.03	Cell membrane
GmCCS17	Glyma.12G081300.1	6423237	6427486	618	20.81	5.79	Nucleus
GmCCS18	Glyma.12G081300.2	6423237	6423451	618	20.58	5.79	chloroplast
GmCCS19	Glyma.12G178800.1	33851671	33854688	636	18.63	6.28	Nucleus
GmCCS20	Glyma.12G210500.1	36949624	36950858	474	16.27	7.67	Nucleus
GmCCS21	Glyma.13G290800.1	39113910	39114984	246	16.20	6.49	Golgi apparatus
GmCCS22	Glyma.16G153900.1	31445530	31449234	396	17.18	7.19	Golgi apparatus
GmCCS23	Glyma.16G153900.2	31445530	31449234	426	16.49	7.19	Golgi apparatus
GmCCS24	Glyma.19G184100.1	44253254	44255354	900	42.28	7.99	Golgi apparatus
GmCCS25	Glyma.19G240400.1	48828599	48831447	927	15.19	5.27	Nucleus
GmCCS26	Glyma.19G240400.2	48827949	48831447	939	13.99	4.86	Nucleus
GmCCS27	Glyma.19G240400.3	48827949	48831447	972	15.19	5.27	Nucleus
GmCCS28	Glyma.19G240400.4	48828007	48831447	330	11.54	5.48	Nucleus
GmCCS29	Glyma.19G240400.5	48827949	48828125	477	13.99	4.86	Nucleus
GmCCS30	Glyma.20G187100.1	42566973	42569993	462	13.56	7.58	Golgi apparatus,Nucleus
GmCCS31	Glyma.20G218500.1	45428471	45432099	1002	20.80	9.41	Nucleus

Supplementary Table S2

>AtCCS1
MAKGVAVLNSSEGVTGTIFFTQEGDGVTTVSGTVSGLKPGLHGFHVHALGDTTNG
CMSTGPHFNPDGKTHGAPEDANRHAGDLGNITVGDDGTATFTITDCQIPLTGPNSI
VGRAVVVHADPDDLGKGGHELSLATGNAGGRVACGIIGLQG

>AtCCS2
MASILRSVATTSAVVAAASAIPIAIAFSSSSSSSSTNPKSQSLNFSFLSRSSPRLLGLSR
SFVSSPMATALTSDRNLHQEDRAMPQLLTEFMVDMTCEGCVNAVKNKL
ETIEGIEKVEVDLSNQVVRILGSSPVKAMTQALEQTGRKARLIGQGVP
QDFLVSAAVAEFKGPDIFGVVRFAQVSMELARIEANFTGLSPGT
HSWCINEYGDLTNGAASTGSLYNPFQDQTGTEPLGDLGTLEADKNGEAFYSGKKE
KLKVADLIGRAVVVYKTDDNKSGPGLTAAVIARSAGVGENYKKLC
SCDGTVIWEATNSDFVASKV

>AtCCS3
MAATNTILAFSSPSRLLIPPSSNPSTLRSSFRGVSLNNNNLHRLQSVSFAVKAPSKAL
TVVSAAKKAVAVLKGTSDVEGVVTLTQDDSGPTTVNVRITGL
TPGPHGFHLHEFGDTTNGCISTGPHFNPNNMTHGAPEDECRHAGDLGNINANADG
VAETTIVDNQIPLTGPNSVVGRAFVVHELKDDLGKGGHELSLTTG
NAGGRLACGVIGLTPL

>GmCCS1
MGEKKEAAKNEGDKKPESGAKQNDGRLPVVLKLDMHCEGCVKKIKRAVRHFDG
VEDVKTD
LSSKKLTVIGKVDPAKVRDKLAEKTKKKVELISPQPKKDSAGDKPPEEKKSEEKKPEDKK
AEEKTPKESTVVLKIRLHCEGCIQKIRKIILKTKGVESVNIEGGKDLVSVKGTMDVKEIV
PYLNEKLKRNVEVVPPKKEGGDKKENNKKEGGGGGGAEGAAKVEVNKMEHYGYAYPAPHM
YWHGHGGYAPGESSSSSSSSNNSYEVEVQSGYSYSNQGYDGNYVNYPYQHGYNDNYMAMA
QPPPPFYLNPHHPPPQMFSDENPNACSVM

>GmCCS2
MVKAVAVLGSSEGVTGTIHFVQEGSGPTTVTGSLAGLKPGLHGFHVHALGDTTNGCLSTG
SHFNPNNKEHGAPEDENRHAGDLGNVNVGDDGTVSFTITDSQIPLTGPNNIIGRAVVVHA
DPDDLGKGGHELSKTTGNAGGRVACGIIGLQG

>GmCCS3
MVKAVAVLGSSEGVTGTIHFVQEGSGPTTVTGSLAGLKPGLHGFHVHALGDTTNGCLSTG
SHFNPNNKEHGAPEDENRHAGDLGNVNVGDDGTVSFTITDSQIPLTGPNNIIGRAVVVHA
DPDDLGKGGHELSKTTGNAGGRVACGIIGLQG

>GmCCS4
MVKAVAVLGSSEGVTGTIHFVQEGSGPTTVTGSLAGLKPGLHGFHVHALGDTTNGCLSTG
SHFNPNNKEHGAPEDENRHAGDLGNVNVGDDDSSHWTKQHHRKGGCCPC

>GmCCS5
MGEEENKPEEPMAKEKNPEEEATQEKKPEQESKEEVAAAAAAPPPPPPPPPPEIVLKVFM
HCEGCARKVRRSLKGFPGVDDVVTDCKSHKVVVKGEKADPLKVLERIQRKSHRQVELLSP
IPKPQEEKKVQEEEKPKPNPEEKKEEPQIVTVLKVHMHCEACSQEIKRRIQRMKGVESAE
PDLKNSQVSVKGVYDPAKLVEYVYKRTGKHAVIVKQEPEKKEKVEEAKEEKKEEEKKSGG
EGEENKEKKEEEAKVEEATTPATTEDTNKVVPEVKINEYFYNPPRYGMEVYAYPAHPAYF

HSYPPQMFSDENPNACTVM

>GmCCS6

MAKEKNPEEEATQEKKPEQESKEEVAAAAAAPPPPPPPPPPEIVLKVFMHCEGCARKVRR
SLKGFPGVDDVVTDCKSHKVVVKGEKADPLKVLERIQRKSHRQVELLSPIPKPQEEKKVQ
EEEKPKPNPEEKKEEPQIVTVLKVHMHCEACSQEIKRRIQRMKGVESAEPDLKNSQVSVK
GVYDPAKLVEYVYKRTGKHAVIVKQEPEKKEKVEEAKEEKKEEEKKSGGEGEENKEKKEE
EAKVEEATTPATTEDTNKVVPEVKINEYFYNPPRYGMEVYAYPAHPAYFHSYPPQMFSDE
NPNACTVM

>GmCCS7

MAFLRSIATTAIATIPAALAFSSSSSSSFPRSSQSPNPQNRLGLVKTLATPPSALHMDHK
LSSQPDAVLPELLTEFMVDMKCEGCVNAVKNKLNEINGVKNVEVDLSNQVVRILGSTPVK
TMTEALEQTGRKARLIGQGVPEDFLISAAVSEFKGPDIFGVVRLAQVNMELARIEANFSG
LSPGKHGWSINEFGDLTRGAASTGKMFNPVNEENSKEPLGDLGTLEANEKGEAFYSGVKE
KLRVADLIGRSVVVYATEDKSEHGITAAVIARSAGVGENYKKLCTCDGTTIWEATDTDFV
TSKV

>GmCCS8

MAFLRSIATTAIATIPAALAFSSSSSSSFPRSSQSPNPQNRLGLVKTLATPPSALHMDHK
LSSQPDAVLPELLTEFMVDMKCEGCVNAVKNKLNEINGVKNVEVDLSNQVVRILGSTPVK
TMTEALEQTGRKARLIGQGVPEDFLISAAVSEFKGPDIFGVVRLAQVNMELARIEANFSG
LSPGKHGWSINEFGDLTRGAASTGKMFNPPLGDLGTLEANEKGEAFYSGVKEKLRVADLI
GRSVVVYATEDKSEHGITAAVIARSAGVGENYKKLCTCDGTTIWEATDTDFVTSKV

>GmCCS9

MTIVEMCVHMDCPGCETKIKKALKKLRGVDDVDIDMRMQKVTVMGWADQKKVLKTVRKTG
RRAELWPYPYNPEYHALARHYGNGNYFASAKPSSSYNYYKHGYSYGEDFGYYHKPIGAAI
IDEKAMSMFSDDNPHACSIM

>GmCCS10

MSSQTVVLKVGMSCQGCAGAVNRVLEKMEGVESFDIDLKEQKVTVKGNVQPDEVLQAVSK
SGKKTAFWVDEAQPPENKPSETAPVTSAENDNKASESGPVASENKPPEAAHVASADPETK
PSETAVETVA

>GmCCS11

MSQTVVLKVGMSCEGCVGAVKRVLGKLDGVESYDIDLKEQKVVVKGNVQPDTVLQTVSKT
GKKTTFWEGEAATSETSTATA

>GmCCS12

MGALDHISELFDCSHTSSKLKKKRKQFQTVEVKVKMDCEGCERKVKKSVEGMKGVTEVEV
DRKASKVTVSGYVEPSKVVSRIAHRTGKRAELWPYLPYDVVAHPYAPGVYDRKAPSGYVR
NADVDPRLTNLARASSTEVKYTTAFSDDNPAACVVM

>GmCCS13

MQLAMAANAVVSPSPFRPQPFLRSSFSGVSVKLTPQSITLSRSKPLTVFAATKKAVAVLK
GTSAVEGVATLIQEDDGPTTVSVRITGLTPGLHGFHLHEYGDTTNGCISTGAHFNPNKLT
HGAPEDEVRHAGDLGNIVANAEGVAEATIVDNQIPLSGPNSVVGRALVVHELEDDLGKGG
HELSLTTGNAGGRLACEGEPWCSGKVVPW

>GmCCS14

MQLAMAANAVVSPSPFRPQPFLRSSFSGVSVKLTPQSITLSRSKPLTVFAATKKAVAVLK
GTSAVEGVATLIQEDDGPTTVSVRITGLTPGLHGFHLHEYGDTTNGCISTAHFNPNKLTH

GAPEDEVRHAGDLGNIVANAEGVAEATIVDNQIPLSGPNSVVGRALVVHELEDDLGKGGH
ELSLTTGNAGGRLACGVVGLTPA

>GmCCS15

MQLAMAANAVVSPSPFRPQPFLRSSFSGVSVKLTPQSITLSRSKPLTVFAATKKAVAVLK
GTSAVEGVATLIQEDDGPTTVSVRITGLTPGLHGFHLHEYGDTTNGCISTGAHFNPNKLT
HGAPEDEVRHAGDLGNIVANAEGVAEATIVDNQIPLSGPNSVVGRALVVHELEDDLGKGG
HELSLTTGNAGGRLACVVGLTPA

>GmCCS16

MQLAMAANAVVSPSPFRPQPFLRSSFSGVSVKLTPQSITLSRSKPLTVFAATKKAVAVLK
GTSAVEGVATLIQEDDGPTTVSVRITGLTPGLHGFHLHEYGDTTNGCISTGAHFNPNKLT
HGAPEDEVRHAGDLGNIVANAEGVAEATIVDNQIPLSGPNSVVGRALVVHELEDDLGKGG
HELSLTTGNAGGRLACGVVGLTPA

>GmCCS17

MQLAVAANAVVSPSPFRPHPLLRSSFSGVSVKLTPQSITFSRLKPLTVFAATKKAVAVLK
GTSAVEGVATLIQEDDGPTTVSVSITGLTPGLHGFHLHEYGDTTNGCISTGAHFNPNNLT
HGAPEDEVRHAGDLGNIVANAEGVAEATIVDNQIPLSGPNSVVGRALVVHELEDDLGKGG
HELSLTTGNAGGRLACGVVGLTPA

>GmCCS18

MQLAVAANAVVSPSPFRPHPLLRSSFSGVSVKLTPQSITFSRLKPLTVFAATKKAVAVLK
GTSAVEGVATLIQEDDGPTTVSVSITGLTPGLHGFHLHEYGDTTNGCISTGAHFNPNNLT
HGAPEDEVRHAGDLGNIVANAEEATIVDNQIPLSGPNSVVGRALVVHELEDDLGKGGHEL
SLTTGNAGGRLACGVVGLTPA

>GmCCS19

MQAAIAAMAAHTILFSSFPAPQSIALPATTKKAVAILKGNSSVHGLVTLTQQQDNGPTTV
TVRVSGLTPGPHGFHLHEFGDITNGCISTGPHFNPNKLKHGAPEDKIRHAGDLGNIVANA
DGVAEATTVDNQIPLIGPNSVVGRALVVHELEDDLGKGGQELSLSTGNAGGRLACGVVGL
SPV

>GmCCS20

MTIIEMRVHMDCPGCENKVKSALQKLKGVDDIEIDMSLQKVTVNGYADQKKVLKTVRKTG
RRAELWQLPYTTDSQNQYVQQHHCNGPINYYASQTSSSYNYYKHGYDSSDPRYYNYPSQS
SIFGYQTGATFSDDNPHACAIM

>GmCCS21

MTIIEMRVHMDCPGCENKVKSALQKLKGVDDIEIDMSLQKVTVNGYADQKKVLKTVRKTG
RRAELWQLPYTTDSQNQYVQQHHCNGPVNYYASQPSSSYNYYKHGYDSSDPRYYNYPSES
SIFGHQTGATFSDDNPDACAIM

>GmCCS22

MEAAKGTVKGVAIIIGDNNIRGSLQFLQHPNGTTHVTGRVTGLSQGFHGFHIHAFGDTTN
GCNSTVLTSSTPGPHFNPFKKDHGAPSDDKRHAGDLGNIAAGPDGVAEISIRDRQIPLTG
VHSIIGRAVVVHADPDDLGRGGHELSKTTGNAGARVACGIIGLQSSV

>GmCCS23

MEAAKGTVKGVAIIIGDNNIRGSLQFLQHPNGTTHVTGRVTGLSQGFHGFHIHAFGDTTN
GCNSTGPHFNPFKKDHGAPSDDKRHAGDLGNIAAGPDGVAEISIRDRQIPLTGVHSIIGR
AVVVHADPDDLGRGGHELSKTTGNAGARVACGIIGLQSSV

>GmCCS24

MGEVSSNTLLYLKTPQYTIHCSVFHESHSLQLSLILFFPFLFLVWQKKEAAKNEADKKPE
SGAKQNDEPVPVVLKLDMHCEGCVKKINRAVRHFEGVEDVKADLSSNKLTVIGKLDPAEV
RDKLAEKTRKKVELVSPQPKKDSAGDKPPEKKTEEKKTEEKKSEDKKAEEKAPKESTVVL
KIRLHCDGCVQKIRKIILKSKGVESVNIEGGKDLVSVKGTMDVKEIVPYLNDKLKRNVEV
VPPKKEGGDNKKENKEGGGGDSKKEGGKKQEGEDGAAKVEVNKMEHYGYGYGYPPPPMYW
YGHGGYAPGESSSYEAEVQPGYNSYSNQGYDGNYGNYHYQGYNNNYMMAQPPPPFYLNPH
HPPPQMFSDENPNACSVM

>GmCCS25

MVKAVAVLGSSEGVTGTIFFTQEGNGPTTVTGSLAGLKPGLHGFHVHALGDTTNGCLSTG
AHFNPNNNEHGAPEDENRHAGDLGNVNVGDDGTVSFSITDSQIPLTGPNSIIGRAVVVHA
DSDDLGKGGHELSKTTGNAGGRVACGIIGLQG

>GmCCS26

MVKAVAVLGSSEGVTGTIFFTQEGNGPTTVTGSLAGLKPGLHGFHVHALGDTTNGCLSTG
AHFNPNNNEHGAPEDENRHAGDLGNVNVGDDGTSDLLFFLLFHLFDLLMSVMMVRLIYYF
FFCFIYLISD

>GmCCS27

MVKAVAVLGSSEGVTGTIFFTQEGNGPTTVTGSLAGLKPGLHGFHVHALGDTTNGCLSTG
AHFNPNNNEHGAPEDENRHAGDLGNVNVGDDGTVSFSITDSQIPLTGPNSIIGRAVVVHA
DSDDLGKGGHELSKTTGNAGGRVACGIIGLQG

>GmCCS28

MVKAVAVLGSSEGVTGTIFFTQEGNGPTTVTGSLAGLKPGLHGFHVHALGDTTNGCLSTG
AHFNPNNNEHGAPEDENRHAGDLGNVNVGDDGGHELSKTTGNAGGRVACGIIGLQG

>GmCCS29

MVKAVAVLGSSEGVTGTIFFTQEGNGPTTVTGSLAGLKPGLHGFHVHALGDTTNGCLSTG
AHFNPNNNEHGAPEDENRHAGDLGNVNVGDDGTSDLLFFLLFHLFDLLMSVMMVRLIYYF
FFCFIYLISD

>GmCCS30

MIYMATFTFSSNDEPYYCSRFIIYVSDFLLCSRPYFPSLPFLKFKTVVLKVGMSCEGCVG
AVKRVLGKLDGVESYDIDLKEQKVVVKGNVQPDTVLATVSKTGKKTTFWEGEAAASETST
ATA

>GmCCS31

MASLLQKAFGSEISSFIYRLFIYQNHNKGIPRNFKMPKKGRPLSLQTVELKVRMCCTGCE
RVVKNAIYKLKGIDSVEVDLEMERVRVGGYVDRNKVLKAVRRAGKRAEFWPYPNPPLYFT
SADHYFKDTTHEFKESYNYYRHGYNLPERHGTMHVSHRGDDNVSNMFNDDNVNACHIM

>ScCCS

MTTNDTYEATYAIPMHCENCVNDIKACLKNVPGINSLNFDIEQQIMSVESSVAPSTIINT
LRNCGKDAIIRGAGKPNSSAVAILETFQKYTIDQKKDTAVRGLARIVQVGENKTLFDITVN
GVPEAGNYHASIHEKGDVSKGVESTGKVWHKFDEPIECFNESDLGKNLYSGKTFLSAPLPTW
QLIGRSFVISKSLNHPENEPSSVKDYSFLGVIARSAGVWENNKQVCACTGKTVWEERKDALANNIK

Supplementary Table S3

GmCCS5-S	GAGGCCAGTGAATTCATGGGAGAGGAGGAAAACAAAC
GmCCS5-AS	ACCCGGGTGGAATTCCTACATGACAGTGCAGGCAT
GmCCS7-S	GAGGCCAGTGAATTCATGGCATTTCTGAGGTCAATAGC
GmCCS7-AS	ACCCGGGTGGAATTCTCAGACCTTGCTAGTAACAAAATCTG
GmCCS8-S	GAGGCCAGTGAATTCATGGCATTTCTGAGGTCAATAGC
GmCCS8-AS	ACCCGGGTGGAATTCTCAGACCTTGCTAGTAACAAAATCTGT
GmCCS11-S	GAGGCCAGTGAATTCATGTCTCAGACTGTTGTCCTC
GmCCS11-AS	ACCCGGGTGGAATTCTTAGGCAGTTGCTGTGCTAGTT
GmCCS24-S	GAGGCCAGTGAATTCATGGGAGAGGTTAGTTCTAATACTCTT
GmCCS24-AS	ACCCGGGTGGAATTCTCACATCACGGAACACGCATTG

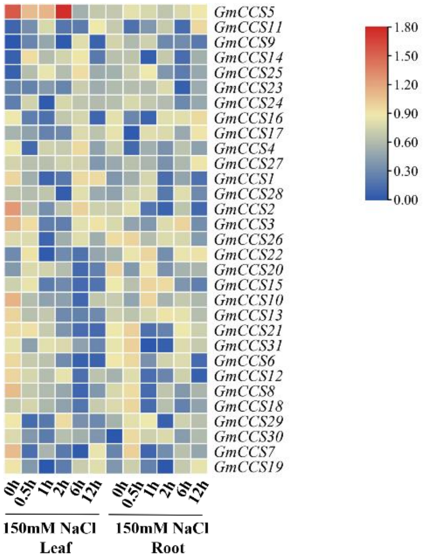

Supplementary Figure S1. RT-qPCR analysis of *GmCCS* genes under NaCl stress. The heatmap was created based on the RT-qPCR results. The transcript abundance is represented by the color bar.

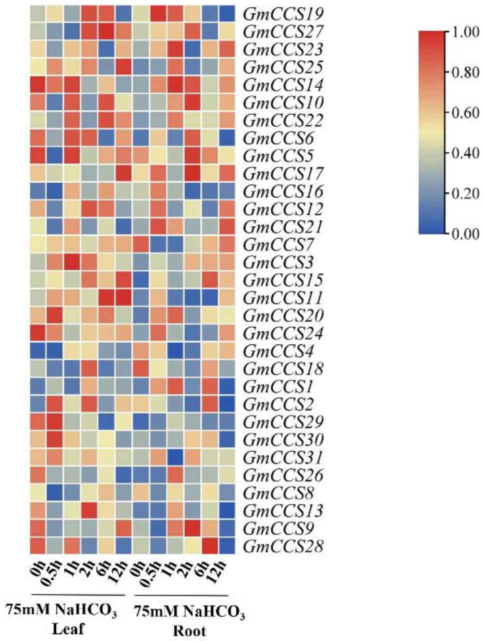

Supplementary Figure S2. RT-qPCR analysis of *GmCCS* genes under $NaHCO_3$ stress. The heatmap was created based on the RT-qPCR results. The transcript abundance is represented by the color bar.

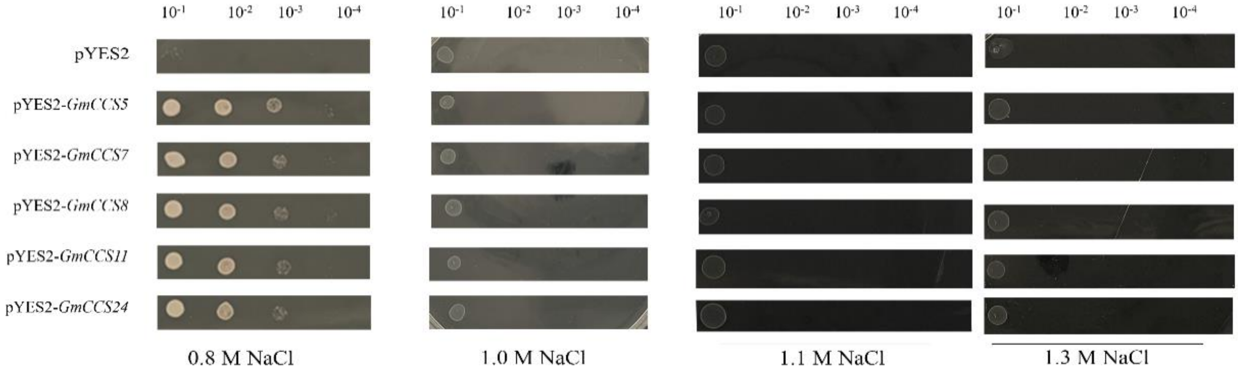

Supplementary Figure S3. NaCl tolerance test of candidate *GmCCS* genes in transformed yeast cells.

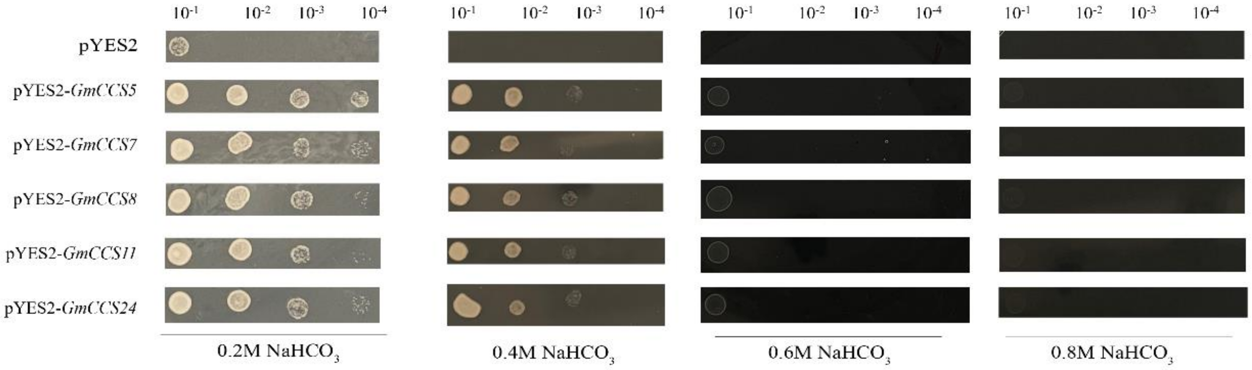

Supplementary Figure S4. $NaHCO_3$ tolerance test of candidate *GmCCS* genes in transformed yeast cells.
